# Effects of different types of fluid resuscitation on hepatic mitochondria and apoptosis

**DOI:** 10.3892/etm.2013.1447

**Published:** 2013-12-12

**Authors:** QINGHONG CHENG, GUANGTIAN YANG, JUANZHEN MA, JIANHUA LI, QI SHAN

**Affiliations:** 1 Department of Intensive Care Unit, First Affiliated Hospital, Medical College, Shihezi University, Shihezi, Xinjiang 832002, P.R. China; 2 Department of Emergency Medicine, Tongji Hospital, Tongji Medical College, Huazhong University of Science and Technology, Wuhan, Hubei 430030, P.R. China

**Keywords:** hemorrhagic shock, fluid resuscitation, mitochondrial membrane potential, succinate dehydrogenase, apoptosis

## Abstract

The aim of this study was to observe the effects of different types of fluid resuscitation on hepatic mitochondria and apoptosis in hemorrhagic shock, and the corresponding mechanisms. Forty rats were divided into five groups: Sham surgery (Sham group), shock (Shock group), Ringer’s lactate resuscitation (RL group), hydroxyethyl starch resuscitation (HES group) and autologous blood resuscitation (BL group). A model of hemorrhagic shock was successfully induced in the latter four groups. The recovery objective was to maintain the mean arterial pressure (MAP) of the rats at 80 mmHg. Two hours after the end of the recovery experiment, fresh liver samples were examined in order to observe the changes in the morphology and mitochondrial membrane potential (ΔΨm). In addition, the levels of succinate dehydrogenase (SDH) activity were assessed, and a terminal deoxynucleotidyl transferase-mediated dUTP nick end labeling (TUNEL) assay was conducted to evaluate the level of apoptosis in the liver cells. In the Shock, RL, HES and BL groups, mitochondrial ultrastructural damage in the liver cells, significant reductions in liver cell function, liver ΔΨm and SDH activity, and the apoptosis of hepatocytes were more apparent compared with those in the Sham group. In the BL group, compared with the RL and HES groups, the injuries to the mitochondrial ultrastructure and liver cell function were significantly reduced, the hepatic ΔΨm and SDH activity were significantly increased and the hepatocyte apoptosis index (AI) was significantly reduced (P<0.05). In conclusion, in a rat model of hemorrhagic shock, different methods of fluid resuscitation may improve the liver cells with regard to mitochondrial ultrastructure and function, the stability of liver ΔΨm, the activity of SDH and the inhibition of liver cell apoptosis. The results indicate that infusion with autologous blood followed by RL solution is a preferable method of fluid resuscitation compared with HES.

## Introduction

The primary treatment for hemorrhagic shock is to control the source of bleeding as quickly as possible and to replace fluid (1). In controlled hemorrhagic shock (CHS), where the source of bleeding has been occluded, fluid replacement is aimed towards the normalization of hemodynamic parameters. In uncontrolled hemorrhagic shock (UCHS), in which the bleeding has temporarily stopped as a result of hypotension, vasoconstriction and clot formation, the aim of fluid treatment is to restore a radial pulse, restore the sensorium or maintain a blood pressure of 80 mmHg with aliquots of 250 ml Ringer’s lactate (RL) solution (hypotensive resuscitation) (2).

When the expected evacuation time is <1 h (usually urban trauma), it is necessary to immediately evacuate the patient to a surgical facility, once the airway and breathing have been secured (3); the introduction of an intravenous line wastes time. When the expected evacuation time is >1 h, an intravenous line is introduced and fluid treatment is initiated prior to evacuation. The resuscitation must occur prior to, or concurrently with, any diagnostic studies (4).

In patients with hemorrhagic shock, hypertonic saline has the theoretical benefit of increasing intravascular volume with only small volumes of fluid (5). The combination of dextran and hypertonic saline may be beneficial in situations where the infusion of large volumes of fluid has the potential to be harmful, such as in elderly individuals with impaired cardiac activity (6). However, additional trials are required before this combination is accepted as a standard of care.

There are recognized risks involved with the transfusion of large quantities of concentrated red blood cells (CRBCs) (7). As a result, alternative modalities are being investigated. One such modality is hemoglobin-based oxygen carriers (HBOCs). The clinical application of the HBOCs has been limited by the toxic effect profile. However, investigations are ongoing into the use of these products (8–10).

Hemorrhagic shock is a common acute and critical illness, and the complication and mortality rates are high (11). The treatment of hemorrhagic shock necessitates the removal of the cause as soon as possible. In addition, timely and effective fluid resuscitation is important (12), in order to improve the oxygen supply to the tissues, and restore the oxygen supply-demand balance and normal cell function. It has been shown that when crystal and colloid droplets are titrated to the same level of filling pressure, they are able to restore tissue perfusion to the same extent (13). However, it has not been elucidated whether the effects of different types of fluid resuscitation on the potential morphological and functional injuries to liver cells during hemorrhagic shock are the same. Apoptosis is a significant form of cell death following ischemia-reperfusion injury. To a certain extent, mitochondrial damage promotes apoptosis. Succinate dehydrogenase (SDH) is an important functional enzyme in the mitochondrial respiratory chain, and measuring the activity of SDH is a method that indirectly reflects the mitochondrial oxidative phosphorylation activity. The reduction of mitochondrial membrane potential (ΔΨm) is considered to be an irreversible event in early apoptosis (14), which occurs before the morphological and biochemical changes in the apoptotic cells. Therefore, inhibiting the reduction in membrane potential may prevent apoptosis.

In the present study a model of hemorrhagic shock was induced in rats, in order to assess the effects of different types of fluid resuscitation on mitochondrial ultrastructure, ΔΨm, SDH activity and liver cell function in rat liver cells. In addition, the development and progression of hemorrhagic shock and the pathophysiological changes in hepatic mitochondria were studied, in order to elucidate the mechanisms underlying the protective effects of different types of fluid resuscitation on liver cells. This may provide an important theoretical basis for clinical treatment.

## Materials and methods

### Animals and grouping

Forty healthy, adult, male Wistar rats, which were supplied by the Shihezi University Laboratory Animal Center (Shihezi, China), were randomly divided into five groups: i) Sham surgery (Sham group, n=8); ii) shock (Shock group, n=8); iii) RL resuscitation (RL group, n=8); iv) hydroxyethyl starch (HES) resuscitation (HES group, n=8); and v) autologous blood resuscitation (BL group, n=8). A comparison of the weights of the rats in the five groups did not reveal any statistically significant differences. This study was performed in strict accordance with the recommendations in the Guide for the Care and Use of Laboratory Animals of the National Institutes of Health (8th edition, 2011). The animal use protocol was reviewed and approved by the Institutional Animal Care and Use Committee (IACUC) of Shihezi University.

### Induction of hemorrhagic shock

All animal experiments were performed in accordance with the National Research Council Ethical Guidelines for the use of animals in and with standard operating procedures (SOP). The anesthetized rats were fixed, the left common carotid artery was isolated, carotid occlusion, proximal, distal occlusion, proximal and distal occlusion in the use of 24G intravenous catheter at the arterial cannulation fixed, the entire pipeline system precharge of heparin saline, carotid artery catheter connected pressure transducer, monitors, continuous hemodynamic monitoring and blood. Similarly the right external jugular vein was isolated for the infusion. Intermittent bleeding until blood pressure stabilized, the rat’s mean arterial blood pressure fell to 40 mmHg, after 1 h shock, group via the right external jugular vein for fluid resuscitation.

### Recovery program

In the Sham group, only the insertion of the arterial catheter was performed, without the bleeding. The Shock group received no fluid resuscitation. Following successful modeling, in the RL group, Ringer’s lactate was infused; in the HES group, the rats were infused with HES 130/0.6 in a sodium chloride injection (Fresenius Kabi Deutschland Gmbh, Beijing, China); in the BL group, the rats were infused with autologous blood, following anticoagulant treatment, and were then infused with Ringer’s lactate. The recovery objective was to maintain the mean arterial pressure (MAP) of the rats at 80 mmHg. Two hours subsequent to the end of the recovery experiment, the rats were sacrificed.

### Specimen collection and observation methods

#### Morphological changes

Samples of fresh liver tissue, measuring ~1 mm^3^, were fixed with 2.5% glutaraldehyde, prior to being dehydrated, embedded, sliced, double-stained and cut into ultrathin sections. Following this, the sections were examined under an electron microscope and the morphological changes in the mitochondria were observed.

#### SDH activity

The liver tissue was homogenized in 0.25 mol/l sucrose solution, pH 7.5. Following this, the homogenate was centrifuged at 1,142 × g for 15 min, prior to the supernatant undergoing further centrifugation at 11,282 × g for 10 min. The mitochondria from the precipitate were subsequently suspended in isolation medium and frozen at −20°C. Twenty four hours subsequent to defrosting, the SDH activity was assessed, in accordance with the kit’s instructions (Kaiji Biological Technology development Co., Ltd., Nanjing, China).

#### Membrane potential

The liver cells (50 μl/100 mg) were suspended in 500 μl JC-1 working solution (Haimen BiYunTian Biotechnology Research Institute, Nantong, China), and a confocal laser microscope (ZEISS LSM510; Germany) was used to observe the changes in membrane potential. In the JC-1 solution there were 50 μl/100 mg cells suspended. The mitochondrial extraction kit was purchased from Haimen BiYunTian Biotechnology Research Institute (number: C3606). The mitochondrial membrane potential detection kit (JC-1) was purchased from Haimen BiYunTian Biotechnology Research Institute (number: C2006). The laser confocal microscope (ZEISS LSM510) was purchased from Carl Zeiss (Thornwood, NY, USA).

#### Pathological changes

The liver tissue was treated using conventional methods to produce paraffin sections, which were then stained with hematoxylin and eosin (H&E). Following this, the pathological changes were observed. The pathological and histological changes were observed under light and the liver tissue pathology integral was calculated.

#### Apoptosis

A terminal deoxynucleotidyl transferase-mediated dUTP nick end labeling (TUNEL) assay (Haimen BiYunTian Biotechnology Research Institute; number C1008) was used to assess the apoptosis of the liver cells. The apoptosis index (AI) was recorded under a light microscope, and apoptotic cells were observed under a fluorescence microscope (CHK optical microscope; Olympus, Tokyo, Japan). The AI was calculated by randomly selecting 10 high-magnification views (x400) under the microscope and, for each high-magnification field of vision, calculating the number of cells positive for apoptosis, per 100 cells. The AI value was expressed as a percentage of positive cells.

#### Statistical analysis

SPSS version 16.0 statistical software (SPSS, Inc., Chicago, IL, USA) was used for the statistical analysis. All measurement data are expressed as the mean ± standard deviation (SD). The overall comparison was performed using analysis of variance (ANOVA), while two samples were compared using the Student-Newman-Keuls (SNK-q) test. The incidence of adverse reactions was compared using the Fisher’s exact probabilities test. The semi-quantitative determination of multiple independent samples (class variables) was performed using the rank sum test. P<0.05 was considered to indicate a statistically significant difference.

## Results

### Electron microscopy

#### Sham group liver tissue

Liver cell blood sinus, the bile duct surface was rich in microvilli and the tight junctions connecting the surface structures were clear. In addition, the membrane structure was normal and the dense cytoplasm was observed to be rich in mitochondria and well organized. The developed endoplasmic reticulum was shown to be rich in glycogen, with few lysosomes and occasional lipid droplets. The nuclei were round and rich in finely granular chromatin.

#### Shock group liver tissue

**Figure 1:**
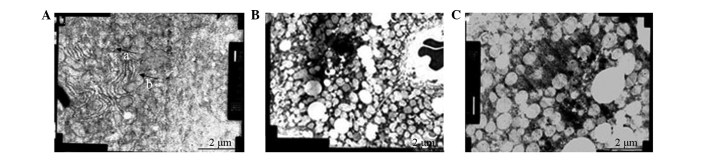
Electron microscopy of the liver. (A) Shock group (magnification, ×10,000); scale, 2 μm. (B) Ringer’s lactate (RL) resuscitation group (magnification, ×30,00); scale, 2 μm. (C) Hydroxyethyl starch (HES) resuscitation group (magnification, ×80,00); scale, 1 μm.

Liver cell blood sinus, the bile duct surface was rich in microvilli and the tight junctions connecting the surface structures were clear. The membrane structure was observed to be normal; however, although the dense cytoplasm was rich in mitochondria, there was an increased volume and a decreased number of mitochondria, and degeneration was apparent. The developed endoplasmic reticulum was rich in glycogen, with few lysosomes; however, there was a slightly increased presence of lipid droplets compared with the Sham group. Rounded nuclei were observed, with fine granular chromatin (Fig. 1A).

#### RL group liver tissue

Liver cell blood sinus, the bile duct surface was rich in microvilli and the tight junctions connecting the surface structures were evident. The membrane structure was normal and the dense cytoplasm was rich in mitochondria. Furthermore, the volume increase was partially attenuated and the degeneration of the mitochondrial cristae was observed to have disappeared. The developed endoplasmic reticulum was glycogen rich, with few lysosomes and it was observed that there was an increased presence of lipid droplets compared with the Sham group. The nuclei were round and rich in finely granular chromatin. The vacuolar area without cell structures showed an uneven distribution of the mitochondrial matrix condensation and there was an uneven distribution of organelles. In addition, a reduction in the number of ribosomes on the rough endoplasmic reticulum, and nucleolar margination was observed, with a greater number of nucleoli (Fig. 1B).

#### Colloid group (HES) liver tissue

Liver cell blood sinus, the bile duct surface was rich in microvilli and the tight junctions connecting the surface structures were clearly visible. The membrane structure was normal, the cytoplasm of mitochondrial centralized, a form of dissolved swelling, and no mitochondrial cristae were present. In the developed endoplasmic reticulum there was an abundance of glycogen, few lysosomes and a significant increase in the number of lipid droplets. The nuclei were round, finely granular and chromatin rich (Fig. 1C).

#### BL group liver tissue

Examination under a microscope revealed a good liver cell morphology, sinusoid surface, and a bile duct surface rich in microvilli. The tight junctions connecting the surface structures were evident. In addition, the membrane structure was normal and the cytoplasm was significantly increased in volume. Furthermore, the deformation of the mitochondria and the degeneration of the mitochondrial cristae were reduced. There was a reduction in the endoplasmic reticulum, glycogen is rich, few lysosomes and a markedly increased presence of lipid droplets. The nuclei were round and rich in finely granular chromatin.

#### Hepatic mitochondrial SDH

Significant differences in the specific activity of mitochondrial SDH were observed between certain groups of rats (P<0.05). The specific activity of SDH in the RL and HES groups was significantly different compared with the Sham group (P<0.05). In the BL group, the activity was reduced compared with the Sham group; however, the difference was not statistically significant (P>0.05). The difference between the BL and Shock groups was significant (P<0.05); furthermore, compared with the BL group, the specific activity of SDH in the mitochondria was significantly different in the RL and HES groups (P<0.05). However, there was no significant difference between the RL and HES groups (P>0.05; Table I).

#### Hepatic ΔΨm

**Figure 2:**
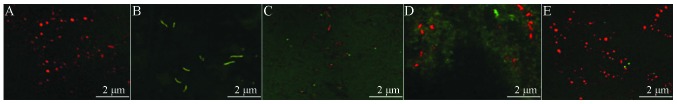
Mitochondrial JC-1 stain images of each group. (A) Sham group (magnification, ×200); (B) Shock group (magnification, ×400); (C) Ringer’s lactate (RL) resuscitation group (magnification, ×200); (D) hydroxyethyl starch (HES) resuscitation group (magnification, ×400); (E) autologous blood resuscitation (BL) group (magnification, ×400). The red/green fluorescence intensity ratio indicated that the level of mitochondrial membrane potential (ΔΨm), decreased the ratio of ΔΨm indicated.

With regard to the ΔΨm of the liver cells, there were statistically significant differences in the RL and HES groups compared with the Sham group (P<0.05). The ΔΨm was reduced in the BL group compared with the Sham group; however, the difference was not statistically significant (P>0.05). There were significant differences in the ΔΨm of the BL and HES groups compared with the Shock group (P<0.05). With regard to the ΔΨm of the three different fluid resuscitation groups, the ΔΨm of the BL and HES groups was significantly different from that of the RL group (P<0.05). No significant difference was observed between the ΔΨm of the BL and HES groups (P>0.05; Table II and Fig. 2)

### H&E staining

#### Sham group liver tissue

**Figure 3:**
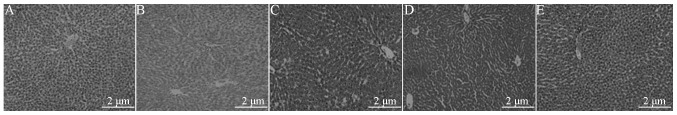
Hematoxylin and eosin (H&E) staining images of the liver. (A) Sham group (magnification, ×100); (B) Shock group (magnification, ×100); (C) Ringer’s lactate (RL) resuscitation group (magnification, ×100); (D) hydroxyethyl starch (HES) resuscitation group (magnification, ×100); (E) autologous blood resuscitation (BL) group (magnification, ×100).

The microscopic examination revealed complete hepatic lobules, with polygonal hepatic cells arranged radially around a central vein, and visible sinusoids neatly arranged into hepatic cords. The basic structure of the hepatic lobule portal area was clearly visible, with no inflammatory cell infiltration (Fig. 3A).

#### Shock group liver tissue

The structural integrity of the hepatic lobules was observed. Low-magnification light microscopy revealed staining of the liver cells, with extensive hydropic degeneration, some congestion of the lobular central vein and sinusoids, vascular dilatation and congestion of the portal area. In addition, the centrilobular portion of the liver denatured, in particular the subcapsule was significant (Fig. 3B).

#### RL group liver tissue

Light microscopy revealed lobular structural integrity, water from the central part of lobule degeneration, the lesion was significantly under the capsule. Vascular dilatation and congestion were observed, in addition to hydropic degeneration of the liver cells and some liver cell necrosis. Furthermore, there was sinusoidal expansion and the infiltration of inflammatory cells, mainly neutrophils (PMNs), under the capsule (Fig. 3C).

#### Colloid group (HES) liver tissue

Light microscopy revealed lobular structural integrity and mild congestion of the central vein and sinusoids. In addition, the focal liver cells were lightly stained. Furthermore, partial liver cell that is nearly subcapsule hydropic degeneration, focal hepatic sinus expansion (Fig. 3D).

#### BL group liver tissue

Light microscopy showed the structural integrity of the hepatic lobules. In the central area of the lobule there was focal hydropic degeneration of the liver cells, in addition to a small amount of subcapsular hydropic degeneration of the liver cells (Fig. 3E).

#### Cell AI

The experimental results revealed that there was a small number of weakly colored positive cells in the Sham group and a large number of deeply colored apoptotic cells in the Shock group. In the recovery groups, it was observed that there was also an increased number of apoptotic cells, mainly around the central vein in the hepatic lobules. In addition, inflammatory necrosis was present in the portal area. The single-factor ANOVA was F=15.755, P=0.000 (P<0.05), which may be considered as the difference between the five groups in general. The pairwise comparison results indicated that the minimum AI was in the Sham group (4.29±3.73), while the highest AI was in the Shock group (42.75±16.42); compared with the three recovery groups, the AIs in the Shock group were statistically significant (P<0.05). With regard to the comparisons among the three different fluid resuscitation group, the AI for the RL (26.63±11.54) and the HES (26.50±8.47) groups was significantly higher than that of BL group (15.25±5.97) (P<0.05). No significant difference was identified between the RL and HES groups (P>0.05; Table III).

#### TUNEL assay

TUNEL-positive apoptotic cells showed small condensed nuclei and a circumscribed nuclear membrane, as the nucleus was stained brown.

#### Sham group liver tissue

**Figure 4:**
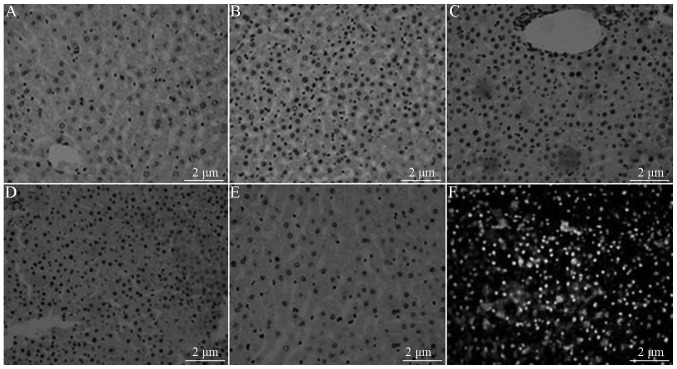
Analysis of liver cell apoptosis in each group by TUNEL staining. (A) Sham group (magnification, ×200); (B) Shock group (magnification, ×200); (C) Ringer’s lactate (RL) resuscitation group (magnification, ×200); (D) hydroxyethyl starch (HES) resuscitation group (magnification, ×200); (E) autologous blood resuscitation (BL) group (magnification, ×200). (F) Fluorescence microscopy of apoptotic cells in liver tissue. TUNEL, terminal deoxynucleotidyl transferase-mediated dUTP nick end labeling.

Under the microscope, a small number of TUNEL-positive apoptotic cells (4.95±5.06)% were observed to be scattered in the liver tissue (Fig. 4A).

#### Shock group liver tissue

When the liver cells were observed under the microscope, extensive hydropic degeneration and a large number of TUNEL-positive apoptotic cells scattered in the liver tissue were observed. Inflammation and necrosis were apparent in a banded and concentrated zone in the portal area. The AI was 73.13±10.51%, which was increased significantly compared with that in the Sham group (Fig. 4B).

#### RL group liver tissue

Under the microscope, an increased number of TUNEL-positive apoptotic cells were observed to be scattered around the central vein and portal area. The AI was 41.88±18.17%, which was significantly less than that of the Shock group (Fig. 4C).

#### Colloid group (HES) liver tissue

Under the microscope, TUNEL-positive apoptotic cells scattered around the blood vessels of the portal area were frequently observed. The AI was 40.25±9.92%, which was significantly reduced compared with that in the Shock group and significantly greater compared with that in the BL group (Fig. 4D).

#### BL group liver tissue

Under the microscope, it was observed that a small number of TUNEL-positive apoptotic cells (4.95±5.06%) were scattered in the liver tissue. Compared with the Shock, RL and HES groups, the number of TUNEL-positive apoptotic cells was significantly reduced (Fig. 4E).

#### Fluorescence microscopy of apoptotic cells in liver tissue

Under a fluorescence microscope, normal cells were stained with light green fluorescence and apoptotic cells were stained yellow-green fluorescence. The apoptotic cells showed nuclear enrichment, nuclear fragmentation and apoptotic bodies, in addition to other withered morphological characteristics of apoptosis (Fig. 4F).

#### Correlation analysis

**Figure 5:**
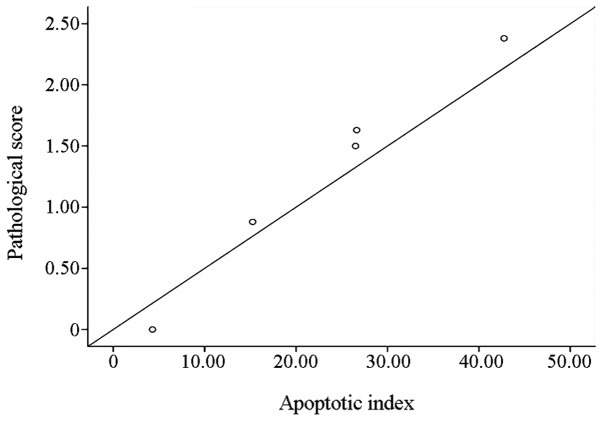
Liver cell apoptosis index (AI) and pathological changes in a linear correlation diagram. The x axis represents AI and the y-axis represents the pathological score. The scatter plot shows that there is a positive linear correlation between the AI and the pathological changes, i.e. as the liver cell AI increased, the pathological change score also increased.

A linear correlation plot was drawn to show the correlation between the liver cell AI and the pathological changes. This is shown in Fig. 5.

## Discussion

In hepatic ischemia-reperfusion, hypoxia-ischemia leads to the production of a large number of oxygen free radicals. The increased presence of oxygen radicals damages the mitochondrial membrane, inhibits membrane protein function and damages nucleotides and other macromolecules, thus undermining the structure and function of the mitochondria (15). The electron microscopy results showed that in the three fluid resuscitation groups, RL, HES and BL, the hepatic mitochondrial injury and the degree of dissolution were reduced, and the amount of damage to the mitochondria was gradually reduced. Resuscitation with HES and blood has been shown to inhibit the body’s inflammatory response, reduce the liver cell and mitochondrial damage following the recovery of rats from hemorrhagic shock and reduce the degree of injury from hepatic ischemia-reperfusion; the recovery following blood resuscitation has been shown to be better than that with HES (16,17). With regard to the changes in mitochondrial morphology, the recovery following resuscitation with autologous blood appears to be better than the recovery following RL and HES resuscitation.

SDH is an enzyme in the mitochondrial respiratory chain. Measuring the activity of SDH indirectly reflects the vitality of the mitochondria (18). The present study showed that there were significant differences between the specific activity of the liver mitochondria SDH in the rats of certain groups. The activities in the Shock, RL and HES groups were significantly lower than that in the Sham group. The SDH activity in the BL group was reduced compared with that in the Sham group; however, the difference was not statistically significant. The mitochondrial activity was higher in the BL group than those in the other two resuscitation groups, indicating that the damage to mitochondrial function and the liver ischemia-reperfusion injury was lower in the BL group than that in the other two resuscitation groups.

ΔΨm is formed from the asymmetric distribution of electrons on the two sides of the mitochondrial membrane. The low permeability of the mitochondrial inner membrane and the electrochemical proton gradient is the basis for maintaining the membrane potential (19). Studies have demonstrated that in the early stages of apoptosis, prior to the onset of pathological changes in the nucleus, the ΔΨm is reduced, suggesting that a decreased ΔΨm is an early stage of apoptosis (17,20,21). The inhibition of the decline in ΔΨm may prevent the occurrence of apoptosis, indicating that cell ΔΨm changes are changes specific to apoptosis (22).

In the present study, it was shown that the hepatic ΔΨm was significantly lower in the Shock, RL and HES groups than in the Sham group. However, although the ΔΨm was reduced in the BL group compared with that in the Sham group, the difference was not statistically significant. The ΔΨm in the BL group was higher than that in the RL and HES groups. The reduction in the ΔΨm of the BL group was smaller than that in the other two groups. It has been suggested that the reduction in ΔΨm is irreversible in early apoptotic events (23,24). With regard to the inhibition of the reduction in transmembrane potential, compared with RL and HES, blood was the ideal recovery liquid and was better able to prevent apoptosis, the reduction in mitochondrial function and the level of liver ischemia-reperfusion injury.

Following traumatic hemorrhagic shock, PMNs and monocytes-macrophages are activated, leading to a broad inflammatory cascade in the body, which is considered to be an important factor leading to injury. The present study showed that with regard to the pathological changes in the liver, the BL group exhibited the minimum pathological liver injury score out of the three recovery groups. The differences between the score in the BL group and the scores in the RL and HES groups, respectively, were statistically significant (P<0.05). This may be due to the fact that RL and HES are only able to increase the circulating blood volume in the body and improve the tissue perfusion in shock: RL and HES do not exhibit an oxygen-carrying capacity, unlike blood resuscitation. As a result of this, RL and HES are not able to significantly attenuate the tissue hypoxia that occurs in shock or reduce the pathological changes to the liver induced by hypoxia.

The results indicated that the AI in the RL group was higher than that in the BL group and significantly lower than that in the Sham group (P<0.05). This result was consistent with the results in the study by Murao *et al*(25). With regard to the effect of RL solution and HES resuscitation on apoptosis, studies have shown that, compared with plasma, whole blood or saline resuscitation, the application of RL solution or HES resuscitation for hemorrhagic shock in rats may significantly increase apoptosis in the liver and small intestine or the lung (25,26). These results were consistent with the results of the present study, in which the AIs of the HES and RL groups were significantly higher than that of the BL group. This may be associated with the fact that the recovery of PMN oxidative burst activity was significantly enhanced (27). However, in the Scultetus recovery with fresh whole blood, increased activation of PMNs was not observed (28). This result was consistent with the fact that the AI for blood resuscitation was the lowest. In the recovery process, the strength of the neutrophil activation is dependent on infusion quantity and speed during the resuscitation (29). PMN as an important effector cells in inflammatory reaction, either in quantity or the content of cytotoxic substance in intra-cytoplasmic in a core position. Although it may kill pathogens by swallow, respiratory burst and degranulation. However, the uncontrolled release of inflammatory mediators may cause systemic inflammatory response syndrome, causing tissue and organ damage and eventually leading to multiple organ dysfunction syndrome. Therefore, PMNs are considered a ‘double-edged sword’ (30), PMN-induced apoptosis occurs mainly through the release of tumor necrosis factor (TNF)-α, interferon (IFN)-7 and interleukin (31), which promotes the increased expression of receptors involved in apoptosis, leading to the apoptosis of hepatocytes (32).

The present study showed that hepatocyte apoptosis and the pathological changes in the liver tissue were positively correlated (r=0.977). This indicated that, in the same group a high liver AI was associated with serious pathological liver injury, although there was some variation in the spatial distribution of the apoptosis and pathological damage. Apoptotic cells more commonly appeared in the lobule around the central veins. With the occurrence of peripheral ischemia in the Shock group, a large number of apoptotic cells were also observed in the hepatic lobule and portal area. A possible explanation for this phenomenon may be that the liver cells around the central vein undergo oxygen exchange, so the apoptosis in this area was connected with hypoxia that caused by so poor blood infusion in pathological conditions.

In this study, how the three types of fluid resuscitation led to apoptosis and why the AIs were different was not elucidated. We propose that it may have been associated with the composition of the resuscitation fluids and the different body stress responses caused by the recovery. Therefore, whereas hepatic ischemia-reperfusion directly leads to apoptosis, the recovery of the cells following RL and HES resuscitation activates a large number of mechanisms and factors associated with apoptosis, unlike blood recovery, which has little effect on these related factors. The specific factors that may induce apoptosis are as follows: i) Recovery of the body following the loss of a large amount of blood causes the body to produce large numbers of oxygen free radicals, which may result in cell apoptosis; ii) blood loss and recovery cause damage to mitochondrial morphology and function, which may stimulate apoptosis; iii) in the blood loss and recovery stress condition, the cells activate self-protective response mechanisms, and endoplasmic reticular stress activates the apoptosis system; iv) following blood loss, hypoxia leads to a decreased production of ATP, and the occurrence of calcium overload also induces apoptosis; and v) recovery of the body following blood loss may lead to the production of a large number of cytokines, and these cytokines may be involved in apoptosis. These factors do not occur singly, but in numerous interactions.

In conclusion, fluid resuscitation following hemorrhagic shock is a complex process. To achieve a recovery effect, it is necessary to perform a comprehensive analysis and a correct diagnosis, in order to avoid complications. The results of this study, with regard to the ultrastructure of the tissue, may be used to evaluate ischemia-reperfusion injury. In the future these results are likely to provide an important reference for fluid resuscitation in hemorrhagic shock.

## Figures and Tables

**Table I:** Effect of different types of fluid resuscitation on hepatic mitochondrial succinate dehydrogenase (SDH).

Group	No. of cases	Enzyme-specific activity (U/mg protein)
Sham	8	167.88±82.49
Shock	8	11.29±18.40^a^
RL	8	29.90±13.29^a^,^b^
HES	8	33.27±12.13^a^,^b^
BL	8	84.70±47.70^c^
F-value		2.754
P-value		0.045

a P<0.05 compared with the Sham group;

b P<0.05 compared with the autologous blood resuscitation (BL) group;

c P<0.05 compared with the Shock group.

RL, Ringer’s lactate resuscitation; HES, hydroxyethyl starch resuscitation.

**Table II:** Effect of fluid resuscitation on hepatic mitochondrial membrane potential.

Group	No. of cases	Membrane potential (red/green)
Sham	27	1.3271±0.6243
Shock	16	0.1519±0.1230^a^
RL	31	0.2816±0.1941^a^
HES	25	0.7786±0.3784^a^-^c^
BL	15	0.8646±0.3222^b^
F-value		6.631
P-value		0.000

a P<0.05 compared with the Sham group;

b P<0.05 compared with the Shock group;

c P<0.05 compared with the autologous blood (BL) resuscitation group.

RL, Ringer’s lactate resuscitation; HES, hydroxyethyl starch resuscitation.

**Table III:** TUNEL assay results for the liver cells in each group.

Group	n	AI
Sham	8	4.29±3.73^a^
Shock	8	42.75±16.42^b^
RL	8	26.63±11.54^b^,^c^
HES	8	26.50±8.47^b^,^c^
BL	8	15.25±5.97^b^
F-value		15.755
P-value		0.000

Results are presented as the mean ± standard deviation.

a P<0.05 compared with the Shock group;

b P<0.05 compared with the Sham group;

c P<0.05 compared with the autologous blood resuscitation (BL) group. TUNEL, terminal deoxynucleotidyltransferase-mediated dUTP nick end labeling.

AI, apoptosis index; RL, Ringer’s lactate resuscitation; HES, hydroxyethyl starch resuscitation.

## Abbreviations

RL: Ringer’s lactate
HES: hydroxyethyl starch
BL: blood resuscitation
MAP: mean arterial pressure
TUNEL: TdT-mediated dUTP nick end labeling
SDH: succinate dehydrogenase
SOP: standard operating procedures
AI: apoptosis index
ΔΨm: mitochondrial transmembrane potential
PMNs: neutrophils
TNF: tumor necrosis factor
IFN: interferon
ATP: adenosine triphosphate
